# Editorial: The microbiome in surgery - friend or foe?

**DOI:** 10.3389/fcimb.2025.1629822

**Published:** 2025-06-25

**Authors:** Ann-Kathrin Lederer, Sophia Chikhladze, Mohamed Tarek Badr

**Affiliations:** ^1^ Center for Complementary Medicine, Department of Medicine II, Medical Center—University of Freiburg, Faculty of Medicine, University of Freiburg, Freiburg, Germany; ^2^ Department of General, Visceral and Transplant Surgery, University Medical Center of the Johannes Gutenberg University, Mainz, Germany; ^3^ Department of General and Visceral Surgery, Medical Center–University of Freiburg, Faculty of Medicine, University of Freiburg, Freiburg, Germany; ^4^ Institute of Medical Microbiology and Hygiene, Medical Center–University of Freiburg, Faculty of Medicine, University of Freiburg, Freiburg, Germany

**Keywords:** microbiota, surgery, postoperative complications, host microbial interaction, immunomodulation, outcome

The human microbiome, an ever-changing consortium of microorganisms found at numerous body sites, plays a pivotal role in health and disease. Surgical interventions, while lifesaving, exert significant physiological stress and can disrupt microbial homeostasis, influencing recovery, infection rates, and overall outcomes.

This Research Topic aimed to elucidate the delicate balance between surgery and the microbiome, highlighting both the beneficial and detrimental impacts of microbes to the surgical environment. The contributions within this Research Topic provide compelling evidence of how microbial communities influence surgical outcomes, and how surgical practices, such as the use of antibiotics and specific procedures, affect microbial balance.

Biesel et al. investigated the impact of microbial colonization in pancreatic cancer on postoperative outcomes following pancreatic surgery. Their findings revealed that specific bacterial profiles within the tumor tissues were associated with a higher risk of postoperative complications, including infections and prolonged recovery. The study highlights the significance of considering intratumoral microbiota as a potential prognostic factor and therapeutic target. Their work contributes to mounting evidence linking microbiome composition to surgical outcomes, especially in oncologic surgery.

Xu et al. reported an intriguing case report of spontaneous bacterial peritonitis development in a malnourished patient with colon cancer due to intestinal barrier dysfunction. The case illustrates how malnutrition and compromised gut integrity in cancer patients can augment the risk to microbial translocation and systemic infection. Their literature review supports a strong interplay between nutritional status, disruption of microbiota, and risk of infection, suggesting that microbiome-based interventions may help prevent complications in such clinical scenarios.

Posadas-Cantera et al. conducted a pilot study to evaluate the diagnostic value of next-generation sequencing (NGS) for diagnosing fungal infection in surgical ICU patients’ ascitic fluid. According to their findings, routine fungal cultures often fail to detect clinically relevant pathogens, while NGS identified a broader and more varied range of fungal species. The study emphasizes the urgent need for advanced molecular diagnostics to improve fungal detection in critically ill patients, particularly when standard methods are insufficient, thereby allowing more targeted antifungal therapy.

Wang et al. explored the the possible link between gut microbiota and postoperative delayed gastric emptying (DGE), a common complication after upper gastrointestinal surgery. Their comprehensive review hypothesizes that microbial dysbiosis, particularly the depletion of short-chain fatty acid–producing bacteria, may affect gastrointestinal motility and inflammation, contributing to DGE. The authors emphasize the need for future studies to investigate targeted modulation of the microbiome as a strategy for prevention or relief of DGE with potentially improved surgical outcomes and patient recovery.

Zhang et al. examined the dental plaque microbiome of alveolar cleft patients to dissect microbial diversity and study potential functional significance in this particular surgical group. Using high-throughput sequencing and functional prediction algorithms, they identified significant microbial composition changes compared to controls, including elevated levels of inflammatory and biofilm-related pathogens. These findings suggest that oral dysbiosis in cleft patients may impair wound healing or increase postsurgical infection risk, suggesting the relevance of microbiome monitoring in oral and maxillofacial surgical planning.

Felgendreff et al. described a novel hypersensitivity reaction characterized by mixed bacterial and fungal dermatitis in a porcine model of non-alcoholic steatohepatitis (NASH). This translational study demonstrates how metabolic liver disease disrupts immune tolerance and microbiome homeostasis to induce hyperactive skin immune responses. The findings offer new insights into the interplay between systemic metabolic disease, skin barrier function, and microbial colonization, and have implications for the treatment of surgical and dermatologic complications in patients with metabolic comorbidities.

Wetzel et al. conducted a longitudinal analysis of gut bacteriome and mycobiome dynamics prior to and following visceral surgery in Crohn’s disease (CD) patients. They identified significant alterations in microbial diversity and community structure, especially in the fungal communities post-surgery. Their results suggest a complex interaction between fungi and bacteria with potential implications for immune response and healing during Crohn’s-related surgery. These findings imply that more research into fungal-bacterial interactions as regulators of surgical outcome is needed.

Loch et al. examined the mucosal microbiome of patients with terminal ileal CD undergoing ileocecal resection. They identified pronounced microbial heterogeneity between inflamed and non-inflamed tissue, revealing spatial variation even within diseased sites. Postoperative microbiome showed partial restoration but remained dysregulated compared to non-CD controls. Their research emphasizes the microbiome’s role in Crohn’s pathogenesis and its potential as a guide for surgical decision-making and therapeutic targeting in inflammatory bowel disease. Collectively, the studies featured in this Research Topic provide valuable insights into the multifaceted interplay between the human microbiome and surgical interventions. These articles emphasize the microbiome’s role not only as a passive bystander but as a dynamic modulator of surgical outcomes, including infection risk, recovery trajectories, and the likelihood of complications. The findings demonstrate the potential of microbiome-based diagnostics and therapeutics such as NGS, microbial biomarkers, and targeted microbiome modulation to revolutionize perioperative care. Importantly, these studies underscore the need to understand microbial dysbiosis in vulnerable patient populations, such as those undergoing oncologic or gastrointestinal surgery, or with underlying inflammatory or metabolic disorders. Moving forward, future research must aim to standardize microbial profiling techniques, establish causal relationships between specific taxa and surgical outcomes, and develop clinical protocols that integrate microbiome management into surgical care pathways. As this field matures, interdisciplinary collaboration between microbiologists, surgeons, and clinical researchers will be critical to translate these findings into routine clinical practice and improved patient care.

